# *N*-Acetyl-d-Glucosamine-Binding Lectin in *Acropora tenuis* Attracts Specific Symbiodiniaceae Cell Culture Strains

**DOI:** 10.3390/md19030146

**Published:** 2021-03-11

**Authors:** Ryota Takeuchi, Mitsuru Jimbo, Fumika Tanimoto, Mariko Iijima, Hiroshi Yamashita, Go Suzuki, Saki Harii, Yoshikatsu Nakano, Ko Yasumoto, Shugo Watabe

**Affiliations:** 1 School of Marine Biosciences, Kitasato University, 1-15-1 Kitasato, Minami, Sagamihara, Kanagawa 252-0373, Japan; r-takeuchi@aist.go.jp (R.T.); pooh.q-.-p.92@docomo.ne.jp (F.T.); m.iijima@aist.go.jp (M.I.); yasumoto@kitasato-u.ac.jp (K.Y.); swatabe@kitasato-u.ac.jp (S.W.); 2 Geological Survey of Japan, National Institute of Advanced Industrial Science and Technology (AIST), 1-1-1 Higashi, Tsukuba, Ibaraki 305-8567, Japan; 3 Fisheries Technology Institute, Japan Fisheries Research and Education Agency, 148 Fukai-Ohta, Ishigaki, Okinawa 907-0415, Japan; hyamashita@fra.affrc.go.jp (H.Y.); gosuzu@fra.affrc.go.jp (G.S.); 4 Sesoko Station, Tropical Biosphere Research Center, University of the Ryukyus, 3422 Sesoko, Motobu, Okinawa 905-0227, Japan; sharii@lab.u-ryukyu.ac.jp; 5 Research Support Division, Okinawa Institute of Science and Technology Graduate University, 1919-1 Tancha, Onna-son, Okinawa 904-0412, Japan; yoshikatsu.nakano2@oist.jp

**Keywords:** *Acropora tenuis*, coral, chemoattraction, lectin

## Abstract

Many corals establish symbiosis with Symbiodiniaceae cells from surrounding environments, but very few Symbiodiniaceae cells exist in the water column. Given that the *N*-acetyl-d-glucosamine-binding lectin ActL attracts Symbiodiniaceae cells, we hypothesized that corals must attract Symbiodiniaceae cells using ActL to acquire them. Anti-ActL antibody inhibited acquisition of Symbiodiniaceae cells, and rearing seawater for juvenile *Acropora tenuis* contained ActL, suggesting that juvenile *A. tenuis* discharge ActL to attract these cells. Among eight Symbiodiniaceae cultured strains, ActL attracted NBRC102920 (*Symbiodinium tridacnidorum*) most strongly followed by CS-161 (*Symbiodinium tridacnidorum*), CCMP2556 (*Durusdinium trenchii*), and CCMP1633 (*Breviolum* sp.); however, it did not attract GTP-A6-Sy (*Symbiodinium natans*), CCMP421 (*Effrenium voratum*), FKM0207 (*Fugacium* sp.), and CS-156 (*Fugacium* sp.). Juvenile polyps of *A. tenuis* acquired limited Symbiodiniaceae cell strains, and the number of acquired Symbiodiniaceae cells in a polyp also differed from each other. The number of Symbiodiniaceae cells acquired by juvenile polyps of *A. tenuis* was correlated with the ActL chemotactic activity. Thus, ActL could be used to attract select Symbiodiniaceae cells and help Symbiodiniaceae cell acquisition in juvenile polyps of *A. tenuis*, facilitating establishment of symbiosis between *A. tenuis* and Symbiodiniaceae cells.

## 1. Introduction

Most reef-building corals establish symbiosis with symbiotic dinoflagellates, which belong to the family of Symbiodiniaceae cells. Symbiodiniaceae cells provide photosynthetic products, which enables corals to effloresce in sub-tropical and tropical regions. When corals lose symbiont cells or photosynthetic pigments of these cells, this event, bleaching, results in coral death [1,2]. Therefore, it is important for corals to symbiose with Symbiodiniaceae cells.

Symbiodiniaceae cells are divided into nine phylogenetically distinct genetic groups (clades A–I) [3,4], and recently, these clades have been proposed as different genera [5]. Each species of coral was reported to have maintained mainly specific genera of Symbiodiniaceae cells [6]. It has been reported that juvenile polyps of *Acropora* sp. acquired *Symbiodinium* and *Durusdinium*, although these genera were rare in the water column [7]. The number of Symbiodiniaceae cells acquired from juvenile polyps of *Acropora tenuis* differed according to the genus of Symbiodiniaceae cells [8,9], suggesting that some selection mechanism exists. Recently, Yamashita et al. reported that juvenile *A. tenuis* attracts some of the genera of Symbiodiniaceae cells during the initial stage of symbiosis [10]. Based on these results, they suggested that *Acropora* corals acquire a few specific genera of Symbiodiniaceae cells at the initial attraction step, followed by subsequent selective uptake by the coral.

Although the mechanism of selective acquisition of Symbiodiniaceae cells is not apparent, some factors for selection have been proposed. It has been reported that artificial green-fluorescent objects under blue LED light attracted free-living Symbiodiniaceae cells [11,12]. On the other hand, Fitt (1981) reported that cnidarians *Aiptasia* sp. and *Cassiopeia xamachana* attracted Symbiodiniaceae cells using ammonium derivatives [13]. In contrast, the planula larvae of *Lobactis scutaria* (referred to as *Fungia scutaria*) attracted these cells by using trehalose, which is also a low-molecular-weight compound [14]. Green fluorescent protein, ammonium derivatives, and trehalose are common substances for corals, and it seems difficult to explain the strict specificity of host–symbiont relationships by these compounds. Recently, Biquand et al. reported that smaller sizes of Symbiodiniaceae cells tend to be acquired by corals more readily [15], but this selection is the last step of Symbiodiniaceae cell acquisition. In the case of *A. tenuis*, it could not explain the acquisition of clades A and D in the larval stage, because they are rare genera in the water column [7].

Nowadays, sugar-binding proteins, lectins, of corals have attracted attention as candidates for the acquisition of Symbiodiniaceae cells. Lectins are involved in symbiosis as well as immunity, development, and differentiation [16,17,18]. In the case of the Nematode *Laxus oneistus*, a C-type lectin, Mermaid, maintained the symbiotic bacteria on their surface, and the subtle differences in their amino acid sequences resulted in the binding of different symbiotic bacteria [19]. In a solitary coral *Lobactis scutaria*, exogenous lectins and some carbohydrates inhibited acquisition of Symbiodiniaceae cells, suggesting that lectins/glycans are involved in the acquisition of Symbiodiniaceae cells in corals. Coral lectins are found in many corals [9,10,16,20], and some of them are involved in coral–Symbiodiniaceae symbiosis. A C-type lectin, Millectin, which was purified from a coral, *Acropora millepora*, bound to bacterial pathogens and Symbiodiniaceae cells [21], and was localized around symbiont cells in the host cells and in nematocysts [22], suggesting that Millectin is involved in immunity and symbiosis. Moreover, a lectin gene, *Pdc*-*Lectin*, found in the coral *Pocillopora damicornis,* was downregulated six days before a bleaching event [23]. These findings suggest the importance of lectins in symbiosis, but their function has not been examined.

Recently, we examined the function of lectins purified from the hard coral *A. tenuis*. *A. tenuis* is a model organism for examining the acquisition of Symbiodiniaceae cells because the planulae of this coral can survive for over a month in glass bottles [24], and they metamorphose easily by treatment with a hydra neuropeptide, Hym-248 [25]. Moreover, metamorphosed juvenile *A. tenuis* polyps acquired particular Symbiodiniaceae cell culture strains containing NBRC102920 (*Symbiodinium tridacnidorum*) [9,10]. We purified two lectins, AtTL-2 and ActL, from *A. tenuis* [10,26]. AtTL-2 is an *N*-acetyl galactosamine (GalNAc)/N-acetyl glucosamine (GlcNAc)-binding lectin and is similar to Tachylectin-2, which was isolated from a horseshoe crab, *Tachypleus tridentatus*. The lectin antibody inhibited the acquisition of the Symbiodiniaceae strain NBRC102920 [10]. Another lectin, ActL, was purified, and its N-terminal amino acid sequence was found to differ from that of AtTL-2 [26]. ActL binds to *N*-acetyl-d-glucosamine (GlcNAc) and shows chemotactic activity toward the Symbiodiniaceae strain NBRC102920. Moreover, GlcNAc inhibits the chemotactic activity of ActL, suggesting that ActL attracts NBRC102920 by binding to Symbiodiniaceae cell-surface carbohydrates. Since cell-surface carbohydrates differ among Symbiodiniaceae strains [27], it is feasible that ActL differentially binds to specific Symbiodiniaceae strains to attract them toward the corals. This may lead to the acquisition of specific Symbiodiniaceae cells by corals. In the present study, we examined whether GlcNAc-binding lectin ActL attracts specific Symbiodiniaceae strains for *A. tenuis* to acquire them.

## 2. Results

### 2.1. Carbohydrate Specificity of ActL

The sugar specificity of ActL was examined using rabbit erythrocytes. The hemagglutination activity of ActL was inhibited by GlcNAc and *N*-acetyl-d-neuramic acid (NANA), and the minimum inhibitory concentrations of GlcNAc and NANA were 1.56 mM and 3.13 mM, respectively.

### 2.2. Participation of ActL in Acquisition of Symbiodiniaceae Cells by Juvenile A. tenuis Polyps

**Figure 1:**
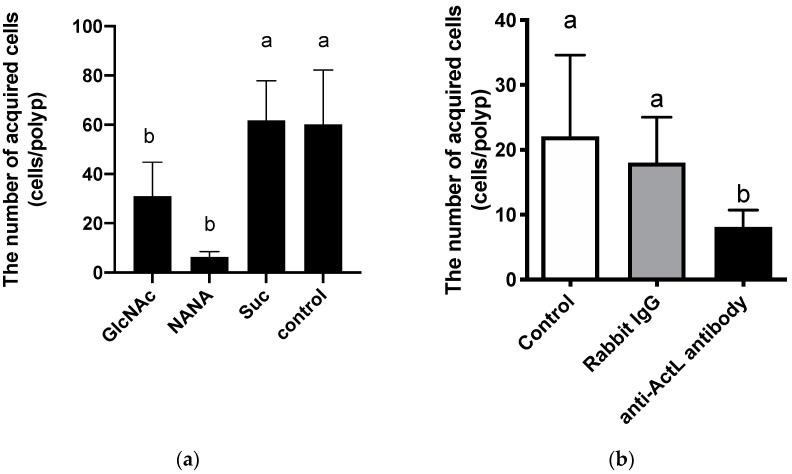
Inhibition of Symbiodiniaceae cell acquisition by carbohydrates or anti-ActL antibody. Juvenile *Acropora tenuis* polyps were incubated at 25 °C with inhibitors for 1 h. Then, polyps were incubated with Symbiodiniaceae NBRC102920 cells for 6 h. The number of Symbiodiniaceae cells in juvenile *A. tenuis* polyps was counted using in vivo chlorophyll a fluorescence. (**a**) Juvenile polyps were incubated with sugars. GlcNAc, NANA, and Suc indicate *N*-acetyl-d-glucosamine, *N*-acetyl-d-neuramic acid, and sucrose, respectively. Control indicates no carbohydrate addition. Values are the mean ± SD (*n* = 4). (**b**) Juvenile polyps were incubated without antibodies, with normal rabbit IgG, or with anti-ActL antibody. Values are the mean ± SD (*n* = 12). Different letters indicate significant differences between antibody treatments (*p* < 0.05, Tukey’s multiple comparisons test).

Previously, we showed that the chemotaxis of Symbiodiniaceae cells to crude extract of *A. tenuis* was inhibited by GlcNAc [26]. To elucidate whether Symbiodiniaceae cell acquisition by polyps was also inhibited by GlcNAc, polyps were incubated with Symbiodiniaceae NBRC102920 cells in the presence of sugars, each at 20 mM concentration (Figure 1a). Symbiodiniaceae cell acquisition was significantly inhibited by GlcNAc (31.0 ± 13.8 cells/polyp, *p* < 0.05) and NANA (6.3 ± 2.2 cells/polyp, *p* < 0.001) compared with that in the absence of sugar (60.0 ± 22.1 cells/polyp) (Figure 1a), suggesting that ActL participates in the Symbiodiniaceae cell acquisition.

Anti-ActL antibody treatment selectively abolished the hemagglutination activity of ActL against rabbit erythrocytes (data not shown). Using this antibody, we examined the participation of ActL on Symbiodiniaceae NBRC102920 cell acquisition. The number of cells acquired by polyps in the presence of the anti-ActL antibody (9.3 ± 5.0 cells/polyp, *p* < 0.05, Tukey’s multiple comparisons test) was significantly reduced compared to that without the antibody (26.3 ± 17.3 cells/polyp) (Figure 1b). These results suggest that ActL participates in Symbiodiniaceae cell acquisition by polyps.

### 2.3. Detection of ActL from A. tenuis Rearing Seawater

**Figure 2:**
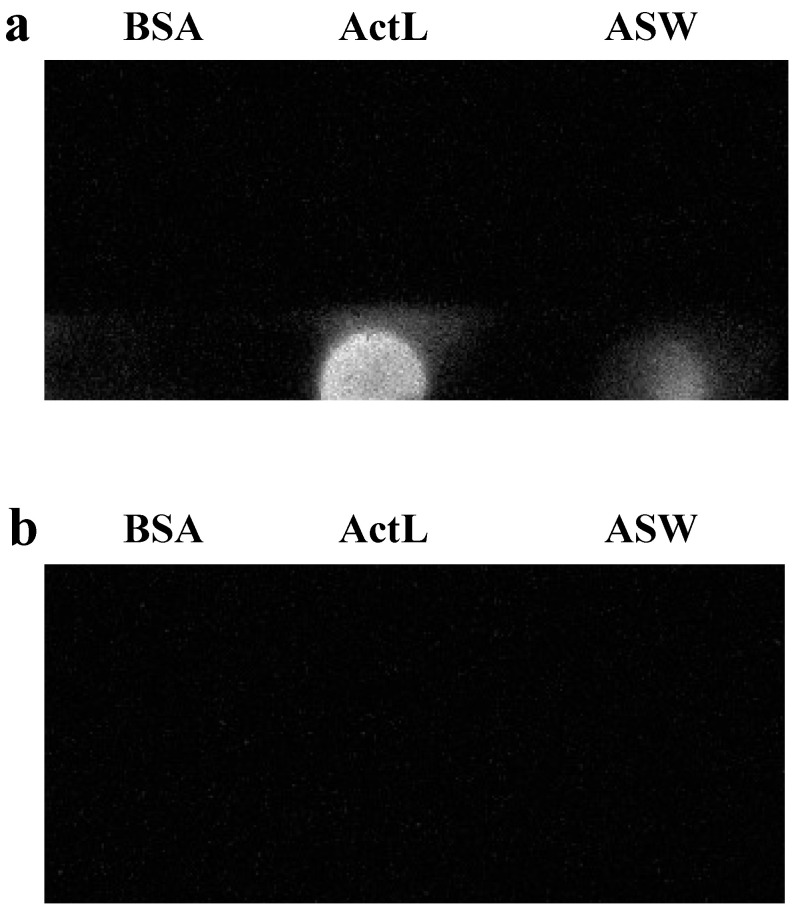
Examination of the occurrence of ActL in rearing seawater of juvenile *A. tenuis* by dot blotting assay. Samples were spotted on the PVDF membrane and detected using the anti-ActL antibody. Samples were 0.1 mg of bovine serum albumin (BSA), 0.1 mg of ActL, and artificial seawater (ASW), where 10 juvenile polyps were kept for 1 d. Binding of the anti-ActL antibody to purified ActL and rearing ASW of juvenile *A. tenuis* polyps was examined. (**a**) Each sample solution dot blotted onto a PVDF membrane. BSA was used as a negative control. Purified ActL was used as a positive control. (**b**) Anti-ActL antibody was preincubated with ActL, and the mixture was used for dot blotting assay instead of the purified anti-ActL antibody.

Using the anti-ActL antibody, we examined by dot blotting, whether ActL was discharged from juvenile *A. tenuis* polyps. Anti-ActL antibody reacted with rearing seawater of juvenile *A. tenuis* polyps (Figure 2a). However, the antibody could also bind to antigens other than ActL, since it was purified using Protein A affinity chromatography. When the anti-ActL antibody was preincubated with ActL and this reaction mixture was used instead of the purified antibody in the experiment, it did not react with rearing seawater (Figure 2b). This indicates that the rearing seawater contained ActL, and juvenile *A. tenuis* polyps discharged ActL outside the body.

### 2.4. Chemotactic Activity of ActL to Each Symbiodiniaceae Strain

**Figure 3:**
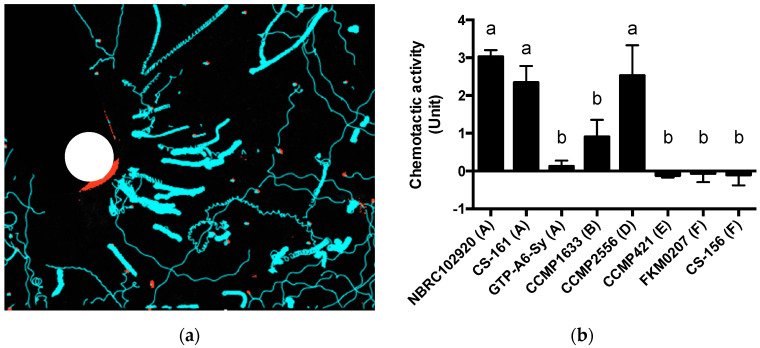
The chemotactic activity of various Symbiodiniaceae strains. (**a**) A capillary containing ActL was placed in NBRC102920 culture in a 35 mm dish. The movements of the Symbiodiniaceae cells were video-recorded. White circle indicates the insertion point of the capillary, red indicates Symbiodiniaceae cells, and cyan lines indicate traces of Symbiodiniaceae cells. (**b**) The chemotactic assay was conducted for 1 h using Symbiodiniaceae cells at a concentration of 1 × 10^6^ cells/mL. One unit (U) of chemotactic activity was defined as the activity at which 40 Symbiodiniaceae cells were attracted by sample solutions in 60 min. Values are the mean ± SD (*n* = 3). Different letters indicate a significant difference in chemotactic activity (*p* < 0.05, Tukey’s multiple comparisons test). The letters in parentheses indicate genera of Symbiodiniaceae; A = genus *Symbiodinium*, B = genus *Breviolum*, D = genus *Durusdinium*, E = genus *Effrenium*, and F = genus *Fugacium*.

It is not known whether and how Symbiodiniaceae cells move toward ActL, although the chemotactic activity assay showed that ActL attracts Symbiodiniaceae NBRC102920 cells [26]. To clarify the chemotactic activity of ActL in detail, the movements of Symbiodiniaceae cells toward ActL were recorded (Figure 3a). The Symbiodiniaceae cells around the capillary moved toward the capillary, whereas the cells distant from the capillary moved linearly in different directions. Since Symbiodiniaceae NBRC102920 cells typically showed circular movement at the same point, this result indicated that ActL attracts Symbiodiniaceae cells.

Next, we measured the chemotactic activity of ActL toward each Symbiodiniaceae strain using chemotactic activity assay [26]. The chemotactic activity was greatest for strain NBRC102920 (3.0 ± 0.2 U), CCMP2556 (2.5 ± 0.8 U), and CS-161 (2.3 ± 0.4 U), followed by CCMP1633 (0.9 ± 0.4 U), GTP-A6-Sy (0.1 ± 0.2 U), CCMP421 (−0.1 ± 0.1 U), FKM0207 (−0.1 ± 0.2 U), and CS-156 (−0.1 ± 0.2 U) (Figure 3). When the chemotactic activity of ActL decreased, the cell movement toward the capillary also decreased (data not shown). This result indicates that the chemotactic activity of ActL varies depending on the Symbiodiniaceae strains. Interestingly, the chemotactic activity is different even in the same genus, but different species.

### 2.5. The Number of Acquired Symbiodiniaceae Cells by Juvenile A. tenuis Polyps

**Figure 4:**
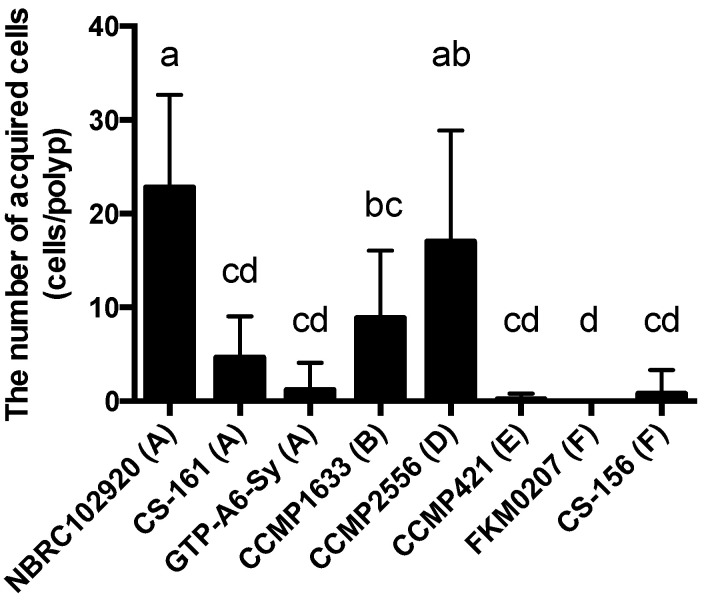
The number of acquired Symbiodiniaceae cells by juvenile *A. tenuis* polyps. Juvenile *Acropora tenuis* polyps were incubated with each Symbiodiniaceae cell strain for 24 h. Symbiodiniaceae cells acquired by juvenile *A. tenuis* polyps were counted using in vivo chlorophyll a fluorescence. Values are the mean ± SD (*n* = 10). Different letters indicate significant differences between Symbiodiniaceae strains (*p* < 0.05, Tukey’s multiple comparisons test). The letters in parentheses indicate genera of Symbiodiniaceae; A = genus *Symbiodinium*, B = genus *Breviolum*, D = genus *Durusdinium*, E = genus *Effrenium*, and F = genus *Fugacium*.

The number of acquired Symbiodiniaceae cells was the largest for NBRC1029020 (22.8 ± 9.9 cells/polyp) and CCMP2556 (17.0 ± 11.9 cells/polyp), followed by CCMP1633 (8.9 ± 7.1 cells/polyp), CS-161 (4.7 ± 4.3 cells/polyp), GTP-A6-Sy (1.2 ± 2.9 cells/polyp), CS-156 (0.8 ± 2.5 cells/polyp), CCMP421 (0.2 ± 0.6 cells/polyp), and FKM0207 (0.0 ± 0.0 cells/polyp) (Figure 4).

**Figure 5:**
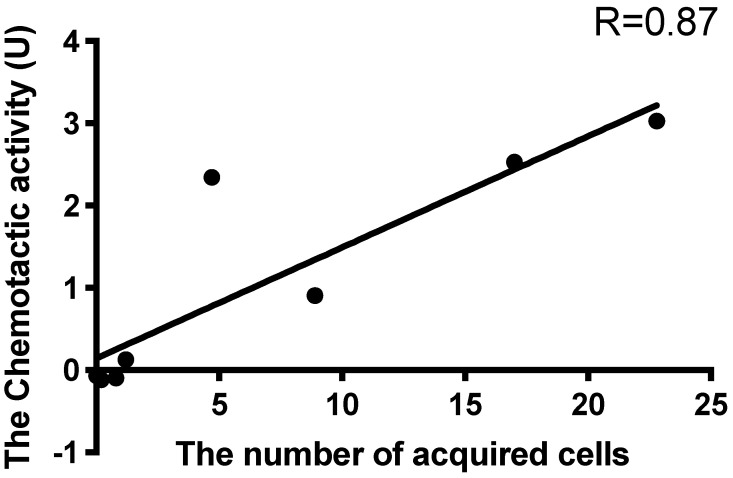
Correlation between the numbers of acquired cells and attracted cells. Pearson’s correlation coefficient was calculated, resulting in a positive correlation (*r* = 0.87).

Next, we explored the correlation between the number of acquired cells and chemotactic activity to examine whether chemoattraction by ActL participates in Symbiodiniaceae cell acquisition (Figure 5). The Pearson’s correlation coefficient showed a positive correlation (*r* = 0.87). The point far from the regression line is a dataset of CS-161. When the point was removed, the Pearson’s correlation coefficient decreased to 0.99.

## 3. Discussion

Corals maintain particular Symbiodiniaceae according to the coral species, and many corals acquire Symbiodiniaceae cells from their surrounding environments. The acquisition process of Symbiodiniaceae cells could mainly be separated into the following two steps: (1) attraction of Symbiodiniaceae cells by corals and (2) phagocytosis of them. Many molecules should participate in this process, but few have been found. We previously purified a GlcNAc-binding lectin ActL from *A. tenuis* [26]. This lectin attracted the Symbiodiniaceae strain NBRC102920, but it was not obvious whether ActL was involved in the acquisition of Symbiodiniaceae cells by the *A. tenuis* polyps. In the present study, we found that the anti-ActL antibody inhibited the acquisition of the Symbiodiniaceae strain NBRC102920 (Figure 1). We also found that GlcNAc inhibited the chemoattraction of ActL [26]. Moreover, rearing artificial seawater (ASW) of *A. tenuis* contained ActL (Figure 2). These results supported our hypothesis that juvenile polyps released ActL to acquire Symbiodiniaceae cells through chemotaxis.

In previous reports, some factors were found to participate in the attraction, including nitrogen-containing compounds such as nitrates and trehalose [13,14]. Many chemotactic compounds are low-molecular-weight substances, and they have the ability to diffuse rapidly and distantly. On the other hand, peptides and proteins, with their high molecular weights, cannot diffuse efficiently and seem not to be attractants. However, some proteins such as startrak have been shown to attract the sperm of starfish [27]. Thus, ActL could be an attractant for Symbiodiniaceae cells. It is possible that ActL could attract Symbiodiniaceae cells in regions close to polyps, since corals live in shallow coral reefs that are exposed to tidal currents and the attractant will be easily diluted. Recently, it has been shown that green fluorescence attracted Symbiodiniaceae cells [12]. Since light could reach a distant location and ActL could become attracted to within a very short range of the polyps, Symbiodiniaceae cells distant from polyps are likely to be attracted by light, and then attracted by ActL later in the process.

A few reports have described the selective attraction of Symbiodiniaceae cells. Blue light may be a factor of selective attraction (Yamashita et al., under review). In this study, Symbiodiniaceae strains were differentially attracted by ActL and *A. tenuis* juvenile polyps. When the number of Symbiodiniaceae strains attracted to ActL was plotted against the number of cells acquired by polyps (Figure 3 and Figure 4), the number of attracted Symbiodiniaceae cells was positively correlated with the number of cells acquired by juvenile polyps (*r* = 0.87, Figure 5). These results indicate that ActL participates in the selection of Symbiodiniaceae strains during acquisition by *A. tenuis* through attraction, except for the Symbiodiniaceae strain CS-161.

CS-161 was highly attracted to ActL, but the number of cells acquired by juvenile polyps was low among the other strains tested (Figure 5). Symbiodiniaceae cell acquisition by juvenile polyps involves several steps, such as attraction and entry into the gastrodermal cells of polyps. Factors related to other steps also affect the number of Symbiodiniaceae cells acquired by juvenile polyps. Indeed, the cell size of Symbiodiniaceae affects the number of cells acquired by juvenile polyps [15]. The factors related to acquisition, except attraction, may inhibit the acquisition of CS-161 by polyps, and this may be a reason why the dataset of CS-161 is an outlier in Figure 5.

Since carbohydrates on the cell surface were different among each strain of Symbiodiniaceae cells [27], ActL could differentially bind to Symbiodiniaceae strains. It was reported that Symbiodiniaceae isolated from the soft coral *Plexaura kuna* had similar lectin binding specificity, although they belonged to different clades [28]. This means that the coral *P. kuna* selectively maintained specific Symbiodiniaceae according to the carbohydrate chains on its cell surface, regardless of clades. The fact that the corals maintained particular genera of Symbiodiniaceae leads to the hypothesis that the selection of Symbiodiniaceae cells by host corals is due to the binding of lectins with Symbiodiniaceae surface carbohydrates. Thus, ActL can attract particular Symbiodiniaceae genera to help polyps to acquire Symbiodiniaceae cells in *A. tenuis* and to maintain particular genera of Symbiodiniaceae. In addition, it is possible that coral colonies also select Symbiodiniaceae cells for maintenance.

On the other hand, Parkinson et al. reported that subtle differences in symbiont cell surface glycan did not explain species-specific colonization rate in the sea anemone, *Exaiptasia pallida* [29]. They showed that lectin masking did not inhibit Symbiodiniaceae cell acquisition by the host *E. pallida*. The paper mentioned that this resulted from the use of adult animals instead of larvae, and the different ages of the animals used. We used different cell densities of added Symbiodiniaceae, while the previously mentioned paper examined the acquisition assay at a very high density of Symbiodiniaceae cells (1 × 10^6^ cells/mL), although Symbiodiniaceae cells at 2000 cells/mL were used in this study. Under high cell density conditions, *A. tenuis* larvae acquire even free-living-type Symbiodiniaceae; however, this was not achieved under low cell density conditions [10]. In our experience, when the density of Symbiodiniaceae cells is very high, Symbiodiniaceae cells will be acquired randomly by hosts (data not shown), and it seems difficult to examine whether Symbiodiniaceae cells will be selectively acquired by the host.

ActL attracted the strains that tend to be acquired; however, there are some conflicts. Although the Symbiodiniaceae strain CS-161 was attracted by ActL at the same level as CCMP2556, CS-161 was acquired less than CCMP2556 by polyps (Figure 5). It was reported that another lectin, *N*-acetyl-d-galactosamine (GalNAc), which is called AtTL-2, participated in *Symbiodinium* acquisition by *A. tenuis* polyps [9]. Since AtTL-2 did not show chemotactic activity in our capillary assay (data not shown), AtTL-2 might participate in other steps of Symbiodiniaceae cell acquisition except attraction, and the low number of acquisitions of CS-161 may be due to the lack of binding of CS-161 to AtTL-2. Light may also be another factor affecting attraction. This indicates that there are other variables that should be identified in the molecular mechanisms of Symbiodiniaceae cells in *A. tenuis*. In this study, we used purified ActL by GlcNAc-affinity chromatography, which is able to bind to AtTL-2. Purified ActL may contain a small amount of AtTL-2. In the future, we must examine whether AtTL-2 participates in chemoattraction and unveil the mechanism of selective chemoattraction by corals.

## 4. Materials and Methods

### 4.1. Materials

*S. tridacnidorum*-cultured strain NBRC102920 (clade A) [30], which was isolated from the giant clam *Tridacna maxima* in Palau, was obtained from the National Institute of Technology and Evaluation (Tokyo, Japan). Cultured strains GTP-A6-Sy (*Symbiodinium natans*) and FKM0207 (*Fugacium* sp.) were originally isolated by Yamashita and Koike (2013) [31]. Cultured strains of CCMP1633 (*Breviolum* sp.), CCMP2556 (*Durusdinium trenchii*), and CCMP421 (*Effrenium voratum*) were obtained from the National Center for Marine Algae and Microbiota (East Boothbay, ME, USA). Cultured strains of CS-161 (*S. tridacnidorum*) and CS-156 (*Fugacium* sp.) were obtained from the Commonwealth Scientific & Industrial Research Organization (Castray Esplanade, Tasmania, Australia). These strains were cultured in IMK medium for marine microalgae (FUJIJFILM Wako Pure Chemical Corporation, Osaka, Japan) at 25 °C with illumination at 80 µmol photons/m^2^/s (12 h:12 h light/dark cycle; 08:00–20:00, light). Unless otherwise specified, all reagents were purchased from FUJIJFILM Wako Pure Chemical Corporation. *A. tenuis* specimens were collected from Sesoko Island, Okinawa, Japan. Collected *A. tenuis* were maintained in an aquarium for several days and then frozen at −70 °C in a freezer until use.

### 4.2. Preparation of ActL

ActL was prepared according to Takeuchi et al. [26]. Briefly, a piece of *A. tenuis* (typically 19 g) was extracted with three volumes of extraction solution (150 mM NaCl, 50 mM Tris–HCl (pH 8.5), and 10 mM CaCl_2_). ActL was purified using a GlcNAc-binding Sepharose 6B column (1 mL) with 0.2 M GlcNAc in extraction solution. The ActL was dialyzed against 1000 volumes of IMK medium at 4 °C for more than 3 h. This process was repeated three times.

### 4.3. Hemagglutination Assay

Purified ActL (20 µL) was serially diluted two-fold using 50 mM Tris–HCl (pH 8.5) containing 150 mM NaCl and 10 mM CaCl_2_ in a V-bottom 96-well microtiter plate. The well was supplemented with 4% rabbit erythrocyte suspension (20 µL) and incubated at 25 °C for 60 min. The titer of the maximum dilution showing positive agglutination was recorded, and the hemagglutination titer was defined as the reciprocal of the highest dilution. Hemagglutination units (HU) were calculated by multiplying the hemagglutination titer by the sample volume.

### 4.4. Carbohydrate Inhibition Test

Purified ActL was diluted to 16 HU using 50 mM Tris–HCl (pH 8.5) containing 150 mM NaCl and 10 mM CaCl_2_. The diluted ActL (20 µL) was incubated for 1 h at 25 °C with diluted carbohydrates. Next, 4% rabbit erythrocyte suspension (20 µL) was added to the mixture, which was then incubated at 25 °C for 30 min, and hemagglutination was measured. The results are expressed as the minimum concentration of carbohydrates. The following carbohydrates were used: l-arabinose, l-fucose, d-ribose, deoxy-d-ribose, d-xylose, d-glucose, d-galactose, l-rhamnose, d-mannose, lactose, melibiose, maltose, d-glucosamine, d-galactosamine, d-mannosamine, *N*-acetyl-d-glucosamine, *N*-acetyl-d-galactosamine, and *N*-acetyl-d-neuramic acid.

### 4.5. Inhibition of Symbiodiniaceae Cell Acquisition by Carbohydrates and Anti-ActL Antibody

Juvenile polyps were prepared as described previously [9]. After 72 h, carbohydrates (GlcNAc, NANA, and sucrose) at 20 mM, rabbit IgG, or anti-ActL antibody (final concentration was 1 µg/mL) was added to ASW-containing juvenile *A. tenuis* polyps, which were then incubated at 25 °C for 1 h before the addition of 2000 NBRC102920 Symbiodiniaceae cells. After an additional incubation at 25 °C for 6 h, the number of Symbiodiniaceae cells within the juvenile *A. tenuis* polyps was counted based on the images obtained by confocal microscopy, as mentioned above.

### 4.6. Dot Blotting Using the Anti-ActL Antibody

Eight wells in a chamber cover grass were filled with 500 µL ASW, and 10 juvenile polyps were kept for 1 d in each of the eight wells. The ASW of 8 wells was then collected, and 30 mL of ice-cold acetone was added and the samples were kept overnight at −35 °C. The sample was centrifuged at 15,000× *g* for 15 min at 0 °C, the supernatant was removed, and the pellet was resuspended in 4 mL of ultrapure water. The solution was subjected to dialysis against 3000 mL ultrapure water at 4 °C for 3 h. This dialysis was repeated three times and the rearing ASW concentrate was obtained. 50 µL of the rearing ASW concentrate and ActL (0.1 mg/mL) were applied to a polyvinylidene difluoride membrane FluoroTrans^®^ W (Pall, Port Washington, NY, USA) using a Bio-Dot apparatus (Bio-Rad Laboratories). After being washed with phosphate-buffered saline (PBS), the membrane was set to a SNAP i.d. 2.0 (Merck Millipore, Bedford, MA, USA), and dot blotting was carried out according to the manufacturer’s protocol with some modifications. The membrane was then blocked with Blocking One (Nacalai Tesque, Kyoto, Japan). Next, it was soaked in 100 μg/mL of anti-ActL antibody diluted using Can Get Signal Solution 1 (TOYOBO, Osaka, Japan) for 10 min and washed with 0.1% Tween-20 in PBS (PBS-T). After that, the membrane was soaked in secondary antibody horseradish peroxidase (HRP)-labeled anti-rabbit IgG antibody (FUJIJFILM Wako Pure Chemical Corporation) in Can Get Signal Solution 2 (TOYOBO) for 10 min. After being washed with PBS-T, the membrane was then transferred onto a wrap and soaked in Luminata Forte Western HRP Substrate (MilliporeSigma, Brington, MA, USA) for 5 min, and the signal was detected using Ez-Capture MG (Atto, Tokyo, Japan). When performing the antibody adsorption test, 1 µg/mL of anti-ActL antibody was incubated with 100 µg/mL of ActL for 16 h. This mixture was then used instead of the anti-ActL antibody. Bovine serum albumin (0.1 mg/mL) was used as the negative control.

### 4.7. Chemotactic Activity Assay

The movements of Symbiodiniaceae cells toward ActL were examined as follows. IMK (2 mL) was added to a 35 mm dish, and a microcapillary containing 2 µL of ActL (100 µg/mL) in IMK medium was added to this dish. After addition of NBRC102920 (1 × 10^4^ cells/mL), the motion around the capillary was video-recorded for 30 s. Symbiodiniaceae cell movements were traced using Particle Track, and analysis (https://github.com/arayoshipta/projectPTAj (accessed on 11 March 2021)).

The chemotactic assay was conducted according to Takeuchi et al. [26]. Briefly, 1.0 × 10^5^ of Symbiodiniaceae cells containing the IMK medium were added to 1.5 mL PROKEEP low protein binding tubes (Fukae Kasei, Kobe, Japan) and subjected to centrifugation at 860× *g* for 5 min at 25 °C. After removing the supernatant, 100 µL of the IMK medium was added to the pellet. The tubes containing pellets were allowed to stand for 24 h to hasten the recovery of Symbiodiniaceae motility, and then the capillaries (Capillary Calibrated Pipettes, Drummond Scientific Company, Broomall, PA, USA) containing 2 µL of sample solutions were inserted into the tubes containing the cells. After 60 min, the capillaries were removed from the tubes, and the solution around the capillary surface was carefully wiped off to remove attached Symbiodiniaceae cells. The solution in the capillaries was blown by mouth into a hemocytometer, and Symbiodiniaceae cells were counted under a microscope. The number of attracted cells was quantified by subtracting the number of Symbiodiniaceae cells in a capillary containing the IMK medium from that containing ActL. The chemotactic activity was calculated according to a standard curve of the number of attracted cells vs. protein concentrations of crude *A. tenuis* extract [26]. One unit (U) of chemotactic activity was defined as the activity at which 40 Symbiodiniaceae cells were attracted by sample solutions in 60 min. Each experiment was performed in triplicate.

### 4.8. Statistical Analysis

All data were analyzed using GraphPad Prism 6.0 for Macintosh (GraphPad Software, La Jolla, CA, USA). Results were analyzed by a one-way repeated-measures ANOVA followed by a Tukey’s multiple comparisons test (*p* < 0.05).
